# Building the blood-brain barrier: a scalable self-assembling 3D model of the brain microvasculature under unidirectional flow

**DOI:** 10.1186/s12987-026-00765-x

**Published:** 2026-01-23

**Authors:** Jade Admiraal, Promise O. Emeh, Marleen Bokkers, Sander P. M. de Ruiter, Thomas Olivier, Karla Queiroz, Todd P. Burton, Nienke R. Wevers

**Affiliations:** 1 MIMETAS BV, Oegstgeest, The Netherlands; 2 Department of Molecular Cell Biology and Immunology, Amsterdam UMC, Location VUmc, Amsterdam Neuroscience, Amsterdam, The Netherlands; 3 Department of Neurosurgery, Brain Tumor Center, Erasmus Medical Center Cancer Institute, Rotterdam, The Netherlands

**Keywords:** Blood-brain barrier, Organ-on-a-chip, BBB-on-a-chip, Unidirectional flow, Perfusion

## Abstract

The blood vessels of the central nervous (CNS) system form a tight, protective blood-brain barrier (BBB). This barrier is essential for healthy CNS function but also poses a hurdle in the treatment of increasingly common neurological disorders. Additionally, BBB dysfunction is a hallmark of many neurological diseases, further emphasizing a need for a better understanding of BBB function in health and disease. We present a human self-assembling 3D model of the BBB in a microfluidic cell culture platform that allows culture of 48 models in parallel on one tissue culture plate. Human brain microvascular endothelial cells, pericytes, and astrocytes form highly reproducible BBB vascular networks under unidirectional perfusion and remain viable for a minimum of 14 days. Immunostaining reveals close cell-cell interactions with pericytes and astrocyte endfeet in direct contact with the brain microvasculature. Compared to endothelial monocultures, co-cultures with astrocytes or pericytes result in improved barrier function, lower vessel diameters, increased branching, and alignment of the vessels in the direction of fluid flow. These results were most pronounced in tri-cultures containing all three cell types. Unlike similar models previously reported, this brain microvasculature model allows for unidirectional perfusion without the need for pumps and syringes. Combined with its high-throughput nature, this feature renders the model suitable for studies of BBB function in health and disease, and assessment of potential BBB restorative therapies.

**Supplementary Information:**

The online version contains supplementary material available at 10.1186/s12987-026-00765-x.

## Introduction

The cerebral blood vessels are formed by highly specialized endothelial cells. These cells are interconnected through tight and adherens junctions, creating a restrictive blood-brain barrier (BBB) [1, 2]. In addition to the endothelial layer, supporting cells such as pericytes and astrocytes play crucial roles in maintaining BBB integrity and function [3–5]. The BBB protects the central nervous system (CNS) by preventing the uncontrolled entry of most circulating molecules and cells, thereby preserving brain homeostasis. Small lipophilic molecules can often cross the BBB via passive diffusion, whereas larger or polar compounds are mostly excluded. Essential molecules that cannot enter the brain via diffusion, enter via active transport mechanisms, such as glucose via the GLUT-1 transporter [4, 6]. In parallel, efflux transporters, such as P-glycoprotein and breast cancer resistance protein 1, actively expel various xenobiotics and drugs, further limiting CNS exposure [7, 8].

While the restrictive nature of the BBB is essential for healthy brain function, it presents a major obstacle for the treatment of neurological diseases. Many therapeutic compounds administered systemically fail to reach the brain in concentrations high enough to elicit a therapeutic response [9, 10]. To address this challenge, various strategies have been developed to improve CNS drug delivery, but effective and predictable delivery across the BBB remains a major hurdle [5, 11, 12].

Disruption of BBB integrity is a hallmark of numerous neurological disorders, including Alzheimer’s disease, Parkinson’s disease, multiple sclerosis, and stroke [13, 14]. In these conditions, increased BBB permeability often coincides with neuroinflammation, exacerbating disease progression. Stroke ranks among the top three global causes of death and as a major cause of adult disability [15, 16]. At the same time, the number of deaths caused by dementias has doubled between 2009 and 2019 [17]. The rising prevalence of such disorders underscores the urgent need for improved understanding of BBB function in both health and disease, as well as for the development of more effective CNS-targeted therapies.

Traditionally, much of our knowledge of the BBB has come from animal models. Although valuable, these models are expensive, time-consuming, and frequently limited by interspecies differences that reduce their translational relevance [18–20]. To address these challenges, human in vitro BBB models have advanced considerably in recent years — evolving from simple monocultures to increasingly sophisticated co-cultures and microfluidic systems that more closely recapitulate the complexity of the in vivo BBB [21]. Microfluidic models comprising endothelial cells, pericytes, and astrocytes show self-assembling BBB vascular networks that allow close cell-cell interactions and lumenized blood vessels [22, 23]. However, the lack of simple approaches for perfused cultured and low-throughput nature of these models poses a hurdle for broader adoption in the field, especially for compound-screening purposes.

Here we present a novel brain microvasculature model, using a microfluidic cell culture platform that enables the scalable generation of self-organized perfused vascular networks [24]. This platform, called the OrganoPlate^®^ Graft 48 UF (Uniflow), harbors 48 chips in a single 384-well titer plate format and allows culture of 48 BBB vascular networks under unidirectional flow on one plate. Primary human microvascular brain endothelial cells (HBMECs) are combined with primary human pericytes and astrocytes and form highly reproducible vascular networks that remain viable for a minimum of 14 days. Dye perfusion shows retention of the dye within the brain microvasculature and bead perfusion reveals unidirectional flow through the vessels, mimicking cerebral blood flow. This model will allow studies of BBB development, its function in health and disease, and assess the potential of BBB restorative therapies.

## Methods

### 2D cell culture

Primary human brain microvascular endothelial cells (HBMEC, ACBRI 376, Cell Systems) were cultured in T75 flasks (F7552, Nunc™ Easy Flask, Sigma) in Promocell MV-2 medium (C-22121, Bioconnect). HBMEC were used for experiments between passage 7 and 8. T75 flasks (F7552, Nunc™ Easy Flask, Sigma) pre-coated with 50 µg/mL poly-L-lysine (3438-200-01, R&D Systems) were used for the expansion of primary human brain vascular pericytes (1200, Sciencell,) and human astrocytes (1800, Sciencell). Pericytes and astrocytes were cultured in their respective media (Pericyte Medium, 1201, Sciencell; Astrocyte Medium, 1801, Sciencell) and used between passage 4 and 5. All cells were maintained at 37 °C, 5% CO_2_ and regularly tested for mycoplasma contamination and found negative. Cells were cultured for 4 days prior to seeding in the OrganoPlate^®^ and dissociated according to supplier’s instructions.

### OrganoPlate Graft 48 UF

**Fig. 1:**
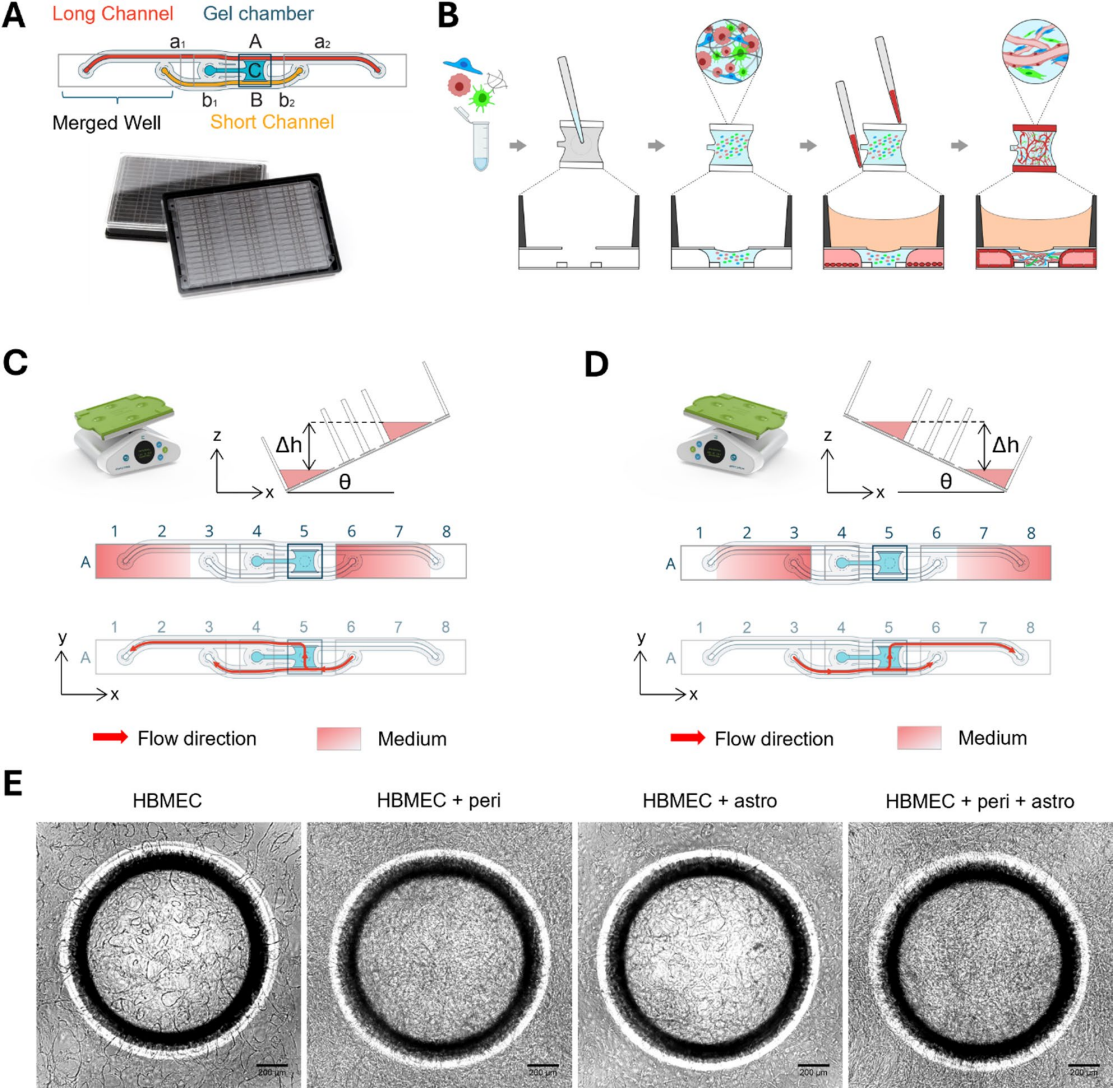
Brain endothelial cells, pericytes, and astrocytes self-assemble into vascular networks in the OrganoPlate Graft 48 UF. (**A**) The OrganoPlate Graft 48 UF is based on a modified 384-well microtiter plate format where long wells are created by merging 3 wells of the titer plate together to link perfusion channels fluidically. It contains 48 chips that can be used to model a BBB vascular network. Each chip comprises a gel chamber (blue) and two perfusion lanes (red, orange). (**B**) Human brain microvascular endothelial cells (HBMECs), astrocytes, and pericytes are embedded in an extracellular matrix gel and seeded to the gel chamber of each chip. Next, HBMECs are seeded in the adjacent perfusion lanes and form vessel-like structures against the extracellular matrix gel. The cells self-assemble into a 3D BBB network. (**C**) Perfusion is generated by placing the OrganoPlate Graft 48 UF on a rocker platform that induces gravity-driven fluid flow. When the angle of inclination is negative, medium is routed through the short channel only and split down two paths: (1) through the gel chamber and towards the long channel (bottom to top of the gel chamber) and (2) continuing through the short channel. (**D**) When the angle of inclination is positive, medium is flowing through the short channel in opposite direction, dividing into similar flow patterns from bottom to top of the gel chamber into the long channel and some flow continuing through the short channel. Red arrow indicates flow direction. (**E**) Phase contrast pictures of BBB monocultures and co-cultures obtained on day 7 after seeding. Scale bar = 200 µm

The OrganoPlate^®^ Graft 48 UF (MIMETAS, Fig. 1A, Suppl. Figure 1) comprises 48 chips. Each chip consists of a gel chamber in which cells embedded in extracellular matrix gel can be added, and two adjacent perfusion lanes. Phaseguides positioned between the gel chamber and perfusion lanes function as capillary pressure barriers to pattern the gel and prevent it from flowing into the adjacent perfusion lanes [25]. Each chip comprises one longer and one shorter perfusion lane that both have inlets and outlets in long wells (3 wells of a 384-well plates merged together). The long wells (Fig. 1C, wells 1–3 and wells 6–8) combined with a low media volume allow for creating an air–liquid interface above the outermost holes in the chip at positions 1 or 8 when the plate is angled above a critical angle. The formation of an air–liquid interface above an inlet hole prevents fluid flow, and the size constraints of the device result in a Laplace value where surface tension prevents the microfluidic channel from emptying. When the angle of inclination is negative, fluid is routed through the short channel and split down the two paths: (1) through the gel chamber and towards the long channel (bottom to top of the gel chamber) and (2) continuing through the short channel. The mode of action is mirrored in the positive incline with media flowing through the short channel in opposite direction, but dividing into similar flow patterns from bottom to top of the gel chamber into the long channel and some flow continuing through the short channel at the bottom (Fig. 1D). During transition from a positive to negative incline or vice versa where all inlets and outlets are covered in fluid there is no pressure difference across the gel chamber and no fluid moved from the short channel towards the long channel.

### OrganoPlate culture

HMBECs, pericytes, and astrocytes were dissociated from flasks and embedded in a mixture of fibrinogen (F3879, Sigma-Aldrich), thrombin (HT1002a, Enzyme Research Laboratories), and Matrigel-GFR (356231, Corning). The resulting gel had a final concentration of 5 mg/mL fibrinogen, 0.2 U/mL thrombin, and 5% (v/v) Matrigel-GFR. HBMECs were seeded at 10,000 cells/µL, pericytes at 2,500 cells/µL, and astrocytes at 500 cells/µL throughout all tested conditions (HBMEC monoculture; HBMEC-pericyte co-culture; HBMEC-astrocyte co-culture; and HBMEC-pericyte-astrocyte co-culture), resulting in a 10:2.5:0.5 HBMEC-pericyte-astrocyte cell ratio as was extensively optimized. 1.5 µL cell-ECM mixture was loaded into the gel chamber of the OrganoPlate^®^ Graft 48 UF (4801400B, MIMETAS). Plates were incubated for 15 min in a humidified incubator (37 °C, 5% CO₂), after which 50 µL of MV-2 medium was added to the gel chamber. A Matrigel-GFR solution (1:100 in PBS, 70013065, Gibco) was then added to the perfusion channels for coating and incubated for 1 h. The coating solution was removed and HBMECs were seeded in the perfusion channels at a density of 10,000 cells/µl using passive pumping technique [26]. Plates were then incubated for 2–3 h (37 °C, 5% CO₂) to allow HBMEC attachment. Next, 100 µL Promocell ECGM-2 medium (C-22011, Promocell) was added to the in- and outlets of both perfusion channels. The medium in the gel chamber was replaced with 50 µL ECGM2 supplemented with 1% pericyte growth supplement (1252, ScienCell) and the plate was placed on an OrganoFlow^®^ perfusion rocker (#MI-OFPR-L, MIMETAS) set at 7° inclination, 8-min interval to media perfusion (bi-directional for first 24 h). One day post seeding, the medium volumes were changed to 40 µL in the perfusion channels and 20 µl in the gel chamber and the plates were placed on an OrganoFlow rocker set at 25° inclination, 1-min interval to allow for unidirectional perfusion to the gel chamber. From day 1–7, the ECGM2 medium added to the perfusion lanes was supplemented with 50 ng/mL VEGF (100 − 20, PeproTech), 250 nM S1P (73914, Sigma-Aldrich) and 100 kIU/mL aprotinin (7005124, Nordic Pharma), while for the gel chamber the ECGM2 was supplemented with aprotinin. After day 7, VEGF and S1P were removed from the medium. Medium changes were performed thrice weekly. The lifetime of the models was not assessed beyond 14 days of culture.

### Immunocytochemistry

Cultures were fixed at day 14 using 3.7% formaldehyde (252549, Sigma-Aldrich) or 100% methanol (-20 °C, 494437, Sigma-Aldrich) and incubated for 15 min. Cultures were permeabilized and blocked for 2 h by adding PBS containing 1% Triton X-100 (T8787, Sigma Aldrich) and 3% bovine serum albumin (BSA, A2153, Sigma-Aldrich). Primary antibodies were prepared in an antibody incubation buffer consisting of 0.3% Triton X-100 and 3% BSA in PBS and incubated at RT overnight. The following primary antibodies were used: mouse anti-PECAM-1 (M0823, DAKO), rabbit anti-VE-cadherin (Ab33168, Abcam), mouse anti-Claudin-5 (35-2500, Thermo Fisher Scientific), rabbit anti-PDGFRβ (Ab32570, Abcam), chicken anti-GFAP (Ab4674, Abcam) and rabbit anti-AQP4 (PA5-53234, Invitrogen). Next, chips were washed 3x with PBS supplemented with 0.3% Triton X-100 (washing solution) and secondary antibodies were applied in antibody incubation buffer and incubated together with NucBlue™ reagent (R37606, Thermo Fisher Scientific) overnight at RT. The following secondary antibodies were used: donkey anti-mouse 488 (A21202, Thermo Fisher Scientific), donkey anti-rabbit 750 (ab175731, Abcam), goat anti-chicken 647 (RA21449, Thermo Fisher Scientific), ActinRed™ 555 reagent (R37112, Thermo Fisher Scientific). Finally, chips were washed with washing solution followed by PBS, after which plates were imaged using the Confocal Micro XLS-C high content imaging systems (Molecular Devices, z-step height 5 μm). Images were post-processed by background subtraction based on the rolling ball radius of 50 pixels.

### Immunostaining quantification

PECAM-1 images obtained from mono- and co-cultures were quantified using Fiji [27]. The images were pre-processed to reduce background signal by applying a rolling ball background correction routine [28]. The center of the gel chamber was selected and segmented using a trainable classifier in Labkit [29, 30]. Vessel signal was extracted as foreground (white), and non-vessel signal was considered background (black). The segmented vessel signal was then skeletonized, after which vascular characteristics were extracted. Vessel area was quantified by measuring the area of vessels and number of branches by calculating the number of branching points of the vessel skeleton. Total vessel length was determined by the sum of all vessel skeleton branches’ length and the average vessel width by dividing the vessel area by total vessel length. Extracted values were converted to appropriate units and then compared across BBB cultures and were plotted using GraphPad Prism, version 10.

### Dye perfusion assay

To confirm the presence of perfusable vessels, 40 µL of ECGM-2 medium containing 0.25 mg/mL FITC-Dextran (150 kDa, 46946, Sigma-Aldrich) was added to the perfusion inlets and outlets, while the gel chambers remained empty. The plates were then placed on the OrganoFlow rocker (25° inclination, 1-min interval) for 4 min to enable dye perfusion through the established vascular network. Fluorescent images were captured at a single timepoint (t = 4 min post dye addition) using an ImageXpress Micro XLS-C system (Molecular Devices) at 37 °C with a 4X objective. This assay was performed at days 7, 9, 11, and 14 of culture.

Dextran perfusion images were pre-processed by limiting the analysis area to the perfusable network. The images were then segmented using IN Carta image analysis software (Version 2.6, Molecular Devices). In short, perfused vessel and non-vessel were identified using a deep-learning model trained on a dataset of similar perfusion images. Using this segmentation, the fluorescence intensity of dye inside and outside the vessels, as well as the surface area of both, were quantified. The data was expressed as a permeability score of sum fluorescence intensity outside the vessels relative to inside the vessels, both corrected for their respective areas.

### Bead perfusion assay

To confirm the presence of perfusable vessels, 40 µL of ECGM2-medium containing 1–5 μm yellow fluorescent polymer microspheres (FMY, Cospheric) was added to perfusion channels inlets and outlets, while the gel chambers remained empty. The plates were then placed on a rocker (set to 25 degrees, 1 min) to enable perfusion through the established vascular network and were monitored using a EVOS™ FL Auto 2 (AMAFD2000, Thermo Fisher Scientific) with a 4X objective. Recordings were captured at 20 frames per second for each of the culture conditions at day 9.

Flow speeds were evaluated by tracking bead velocities using Fiji and estimated for four representative vessels differing in size and location within the vascular network. The analysis focuses on a small vessel (1), a large vessel (2), the main perfusion tubule (3), and its connecting branch linking to the surrounding microvasculature (4) of the co-culture of HBMECs, pericyte and astrocytes.

The recordings were pre-processed by using a Kalman filter [31] to reduce high gain noise from the time lapse recording and used for bead perfusion quantification. The angular local orientations of the bead flow were then quantified using OrientationJ [32–34]. The visual directional analysis was used to create example images of each culture condition, overlaying local orientations as colors (hue) on the original image. The vector field implementation was used to quantify the local orientations and was measured for each frame of the recording, and post-processed using Python.

### P-glycoprotein functionality

P-glycoprotein (P-gp) assays were performed as previously described [35]. In short, calcein-AM (0.5 µM, C3099, ThermoFisher), a substrate of P-gp, was added to all inlets and outlets and graft chambers of chips in presence or absence of P-gp inhibitor cyclosporin-A (10 µM, 30024, Sigma) and incubated for 2 h. Next, the reaction was stopped by perfusing cold medium with cyclosporin-A and Hoechst (H3570, ThermoFisher) to stain the nuclei. Z-stacks (5 μm step size) were acquired using the ImageXpress^®^ Micro Confocal High Content Imaging System (Molecular Devices). Maximum projection images were used to normalize green-fluorescent calcein signal intensity to Hoechst cell count to quantify the intracellular calcein in conditions with and without P-gp inhibitor using IN Carta image analysis software (Version 2.6, Molecular Devices).

### Statistical analysis

**Fig. 2:**
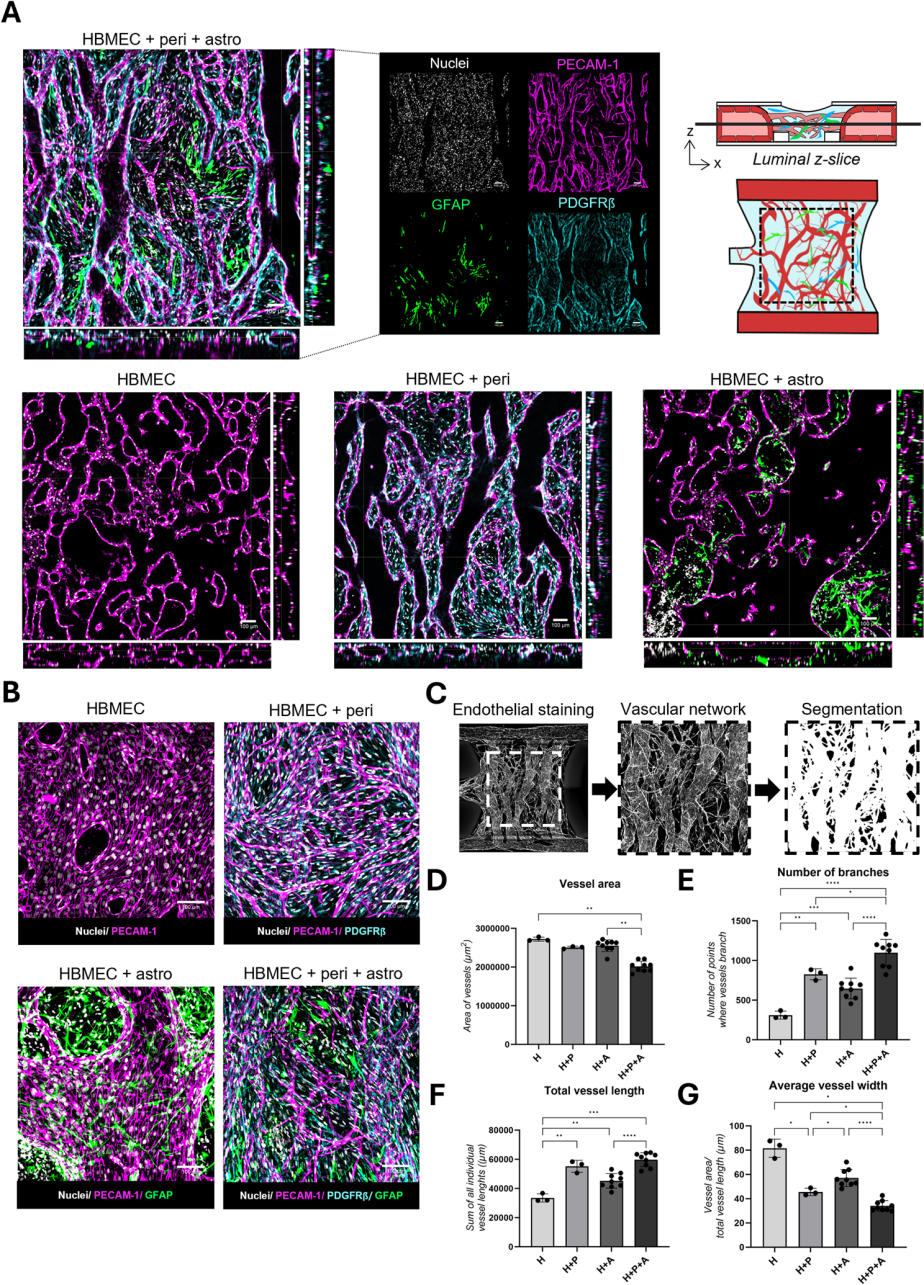
Vascular networks show brain endothelial vessels in close contact with pericytes and astrocytes. (**A**) Cerebral vascular networks consisting of (1) HBMECs (2), HBMECs + pericytes (3), HBMEC + astrocytes, or (4) HBMECs + pericytes + astrocytes) were cultured for 14 days and stained for nuclear marker (gray), endothelial cell marker PECAM-1 (magenta), pericytic marker PDGFRβ (cyan), and astrocytic marker GFAP (green). Orthogonal projections display single-slice merged images as indicated in the illustration of the top view, front view, and side views of the gel chamber. For co-culture of HBMECs, pericytes and astrocytes a merged image as well as the separate markers are shown. Scale bar = 100 μm. (**B**) High magnification maximum projection merged images of the different BBB culture setups. Scale bar = 100 μm (**C**) Maximum projection images of the vasculature marker (PECAM-1) on day 14 were used for quantification purposes following a segmentation procedure. (**D**) Quantification of vascular network area. (**E**) Quantification of total number of branches. (**F**) Quantification of total vessel length. (**G**) Quantification of average vessel width of different BBB cultures. Data in D-G represents the mean ± SD for *n* = 3–9 chips, *(*P* < 0.05), **(*P* < 0.01), ***(*P* < 0.001), ****(*P* < 0.0001)

**Fig. 3:**
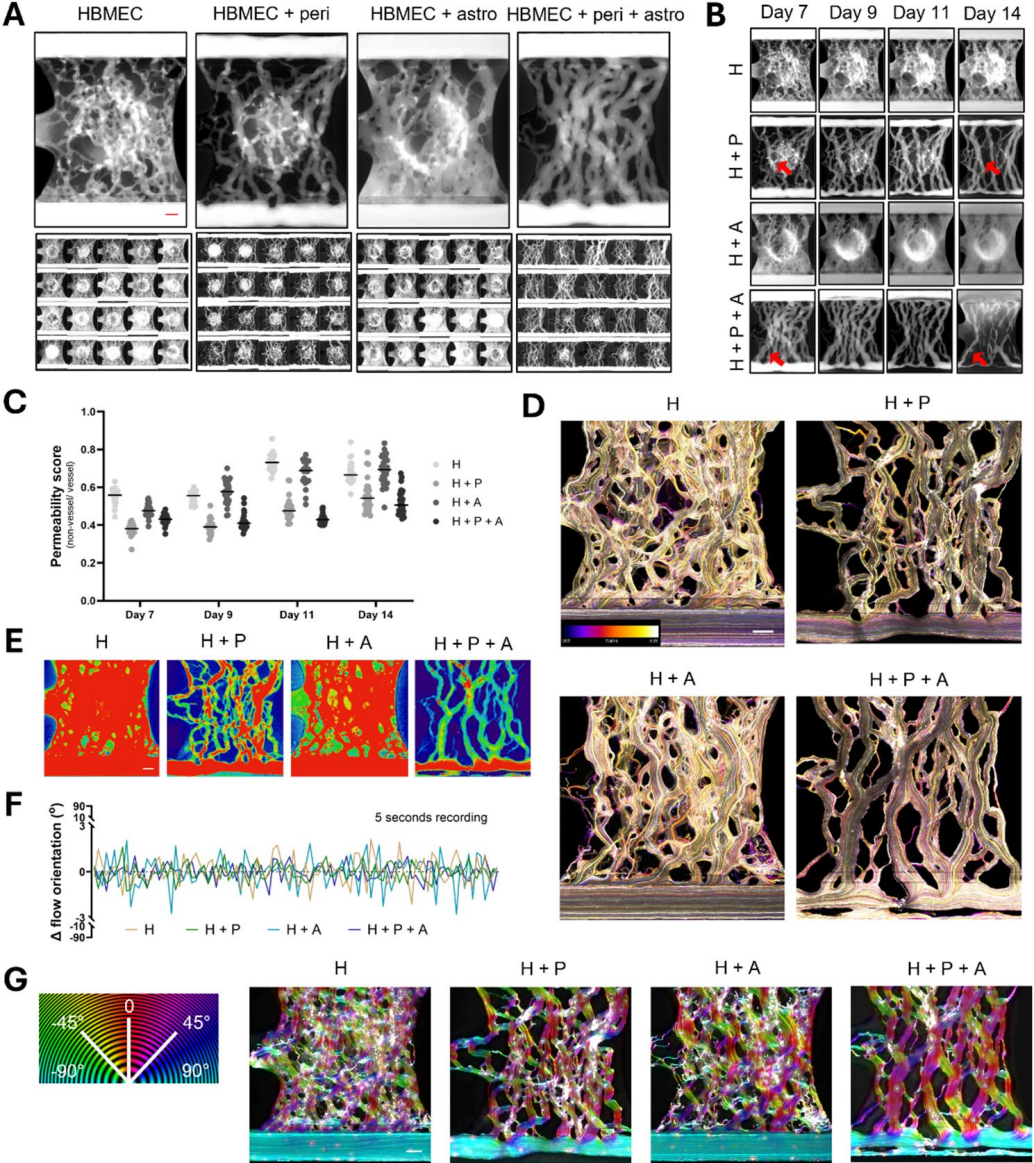
Robust brain microvasculature formation and perfusion. (**A**) 150 kDa FITC-dextran dye was perfused through BBB vascular networks consisting of (1) HBMECs (2), HBMECs + pericytes (3), HBMECs + astrocytes, and (4) HBMECs + pericytes + astrocytes. Upper panels show representative images for each culture condition obtained 4 min after dye addition. Lower panels show a montage of 20 assessed chips for each condition at day 7 of culture. (**B**) Representative images showing perfusion of the different BBB vascular networks with a 150 kDa FITC-dextran dye was assessed at different time points (day 7–14) (representative images per condition of *n* = 20). Arrows indicate areas that show vascular remodeling and pruning over time. (**C**) Permeability score of 150 kDa FITC-dextran dye between non-vessel area and vessel area for the different BBB co-culture setups assessed at different time points (day 7–14). Data represents individual data points and the median for *n* = 21–29 chips. For visualization of statistical significance, see supplementary Fig. 4A. (**D**) Fluorescent beads were perfused through the cerebral vascular networks. Bead flow was captured via high-speed fluorescent imaging. Colors correspond with frame number in the color-coded image indicated in the colormap. (**E**) Heatmaps show low-traffic (blue) and high-traffic (red) bead flow areas in BBB vascular networks consisting of (1) HBMECs (2), HBMECs + pericytes (3), HBMECs + astrocytes, and (4) HBMECs + pericytes + astrocytes. (**F**) Change in flow orientation over time. A value close to 0 means limited directional changes are found relative to the previous frame, and values deviating from 0 mean significant angular changes compared to the previous frame. (**G**) Flow directions were color coded for each culture setup, with flow from right to left (-90°) and left to right (90°) represented in teal and flow straight through the gel chamber (0°) depicted in red. White colors imply no direction can be determined for that location. Scale bar = 200 μm

Data was analyzed using GraphPad Prism (Version 10.6.1). Gaussian distribution for immunostaining quantification was assessed using the Shapiro-Wilk normality test, for *n* = 3–9 replicates. Equality of variances was assessed using the Brown–Forsyth. A Brown-Forsythe and Welch test including a Dunnett T3 multiple comparisons test was performed in case of normally distributed data in which the assumption of equality of variances was violated (Fig. 2E-G). When normal distribution could not be confirmed (Fig. 2D), the nonparametric Kruskal-Wallis test with Dunn’s multiple comparisons test was performed. Statistical significance was indicated by one or more asterisks. *(*P* < 0.05), **(*P* < 0.01), ***(*P* < 0.001) or ****(*P* < 0.0001). Normal distribution of permeability data (Fig. 3C) was confirmed using a QQ plot and homogeneity of variances by a homoscedasticity plot, for *n* = 21–29 chips. A two-way ANOVA was performed and pairwise comparisons between experimental conditions within each timepoint were adjusted for multiple testing using Tukey’s honestly significant difference procedure. Statistical significance was visualized by plotting Tukey-adjusted 95% confidence for pairwise differences (Suppl. Figure 4 A).

## Results

### Brain endothelial cells, pericytes, and astrocytes self-assemble into vascular networks in the OrganoPlate Graft 48 UF

We employed a novel 3D cell culture system, called the OrganoPlate Graft 48 UF. This plate consists of 48 microfluidic chips in a 384-well microtiter plate format (Fig. 1A, Suppl. Figure 1). The OrganoPlate Graft 48 UF chip design includes a graft chamber lined by a long and a short channel. Primary human brain microvascular endothelial cells (HBMECs), pericytes, and astrocytes were embedded in an extracellular matrix (ECM) gel consisting of fibrin and Matrigel and loaded into the gel chamber of each chip. After gelation, HBMECs were added to the two adjacent perfusion lanes (Fig. 1B).

Perfusion of the cultures is gravity-driven and established by placing the OrganoPlate Graft 48 UF on a rocker, which generates a pressure gradient through the vessels enabling continuous perfusion throughout the 14-day duration of the culture. Unidirectional flow is achieved by the creation of a pressure difference between the long and short channels at both negative and positive rocking angles (Fig. 1C–D). At both negative and positive inclines (Fig. 1C-D), medium flows from the short perfusion lane through the gel chamber toward the long perfusion lane. This ensures a consistent bottom-to-top perfusion through the gel chamber due to the pressure differences between the perfusion channels, enabling sustained unidirectional flow across the vascular network. However, due to the self-organization of the vascular structures, there are instances of a short period of bidirectional or static flow if the micro vessels are interconnected along the length of a tube, this is limited up to 8 s on average compared to 52–60 s of unidirectional flow. This results in the formation of majority unidirectional perfused ECM embedded BBB vascular network connected to two larger endothelial vessels (Fig. 1E).

### Vascular networks show brain endothelial vessels in close contact with pericytes and astrocytes

Cerebral networks consisting of (1) HBMECs only (2), HBMECs and pericytes (3), HBMEC and astrocytes, or (4) HBMECS, pericytes, and astrocytes were cultured in the OrganoPlate Graft 48 UF for 14 days and characterized using immunofluorescent staining. Confocal imaging revealed hollow structures lined by PECAM-1 positive cells, indicating a 3D network of lumenized endothelial vessels, in all tested culture setups (Fig. 2A-B, Suppl. Figure 2C, Suppl. Video 1–4). In addition, the vessels showed expression of endothelial marker VE-cadherin as well as tight junction protein Claudin-5 (Suppl. Figure 2A), confirming the junctional phenotype specific to the BBB. Pericytic marker PDGFRβ is observed on the outside of the endothelial vessels, directly in contact with the endothelial cells, as are the endfeet of astrocytes, expressing GFAP (Fig. 2A-B). The interaction between astrocytes and endothelial vessels was further confirmed by the expression of the water channel Aquaporin-4 (AQP4) in close proximity to the endothelial marker PECAM-1 (Suppl. Figure 2A, Suppl. Figure 3). A combination of distributed AQP4 expression across astrocytes and a more localized expression at the astrocytic endfeet was observed. To further characterize the vascular networks, PECAM-1 staining images from day 14 were converted into binary images in which white signal indicated the presence of vessels with their branches (Fig. 2C, Suppl. Figure 2B). A significant reduction in vascular network area was observed with the addition of pericytes and astrocytes compared to HBMEC monocultures (*P* = 0.0017 for triculture versus monoculture) (Fig. 2D). Additionally, the brain microvasculature of BBB co-cultures showed a significant increase in vascular branching compared to HBMEC monocultures, especially in the tricultures (1098 branching points vs. 312 branching points, *P* ≤ 0.0001) (Fig. 2E). Finally, addition of pericytes and astrocytes resulted in a significant increase in total vessel length (*P* = 0.0001) and a reduction in average vessel width compared to HBMEC monocultures (34 μm in tri-cultures vs. 82 μm in monocultures, *P* = 0.0272) (Fig. 2F-G). Together, these data show more intricate microvascular network formation in the presence of astrocytes and pericytes.

### Robust formation and perfusion of cerebral vascular networks

To confirm that the hollow vessel structures as observed via immunostaining were functional, the cerebral vascular networks were perfused with a fluorescent dye from day 7 onwards. A minimum of 20 chips were assessed per culture condition at day 7, 9, 11, and 14 after cell seeding. Perfusion of a fluorescent dextran dye through the BBB vascular networks showed retention of the dye in the vessels’ lumens with high reproducibility (Fig. 3A). All BBB vascular networks models showed robust perfusion (> 95% chips) over the assessed period of day 7–14 of culture (Fig. 3B). A permeability score that reflects the dye signal outside and inside the vascular networks was used as to compare the conditions’ relative barrier function. HBMEC monocultures and co-cultures of HBMECs with astrocytes show slightly reduced barrier function as confirmed by an increase in permeability score across all chips. In contrast, vascular networks containing pericytes showed minimal leakage of dye into the ECM gel, resulting in significantly lower permeability scores (Fig. 3B-C, Suppl. Figure 4 A). A small increase in permeability for the co-cultures with pericytes and astrocytes is found on day 14 compared to earlier days. Additionally, pericyte-containing co-cultures show vascular remodeling and pruning of the network as well as contraction of the two larger vessels in the perfusion lanes, likely due to the contractile nature of the pericytes.

In addition, flow direction through the vascular networks was assessed via perfusion with fluorescent microsphere beads of 1–5 microns in size. Beads were added to the perfusion lanes and their flow through the chips was monitored via high-speed fluorescent imaging at day 9 of the culture. The fluorescent beads reveal a unidirectional flow through the BBB vascular networks, entering from the bottom perfusion lane and exiting via the top perfusion lane (Suppl. Videos 5–8, Fig. 3D). Along with flow direction, flow speed was evaluated by tracking bead velocities in vessels of varying sizes (Suppl. Figure 4B). Beads in small capillaries exhibited flow speeds ranging from ~ 0.1 to 2.6 mm/s, with larger vessels exhibiting velocities up to ~ 3.8 mm/s. The velocity in the main perfusion tubules and interconnection of vessels can be much higher with a maximum measured speed of ~ 18 mm/s. However, several factors can influence flow speed including vessel diameter and rocker configurations. High- and low-bead traffic areas were visualized using a heatmap approach (Fig. 3D) and show higher bead traffic in networks devoid of pericytes, in line with the higher vessel width and lower vascular branching observed in these conditions (Fig. 2D-E). Quantification (Fig. 3F) and color-coded visualization (Fig. 3G) of bead flow confirmed that the overall flow patterns through the vascular networks are unidirectional in nature for all BBB models. More detailed assessment revealed that beads perfused through HBMEC monocultures and co-culture of HBMECs and astrocytes showed more variation in flow direction compared to those perfused through cultures containing pericytes, which show alignment of the vessels in the direction of fluid flow. This result was most pronounced in tri-cultures containing all three cell types.

Finally, the function of P-gp, an essential efflux transporter at the BBB [36], was assessed by adding the fluorescent P-gp substrate Calcein-AM and monitoring intracellular fluorescence in the absence and presence of a P-gp inhibitor. In a proof-of-concept experiment, BBB co-cultures co-incubated with P-gp inhibitor cyclosporine A [36] showed an approximately two-fold increase in intracellular calcein signal compared to cultures incubated with calcein-AM alone, providing preliminary evidence for P-gp activity (Suppl. Figure 5).

## Discussion

In this study, we present a 3D self-assembling blood–brain barrier (BBB) model comprising primary human brain endothelial cells, pericytes, and astrocytes cultured under gravity-driven unidirectional flow. Immunostaining revealed a network of endothelial vessels in direct contact with pericytes and astrocyte endfeet, embedded in ECM gel. The endothelial networks consist of functional vessels as evidenced by the perfusion with fluorescent dye and beads. Using a novel chip design, we show unidirectional flow through the BBB networks without the need for pumps or syringes.

A key focus of this work was to investigate the influence of pericytes and astrocytes – separately and in combination – on the formation, organization, and perfusion of the BBB vascular network. All tested culture configurations, including (1) HBMEC monocultures (2), HBMEC + pericyte co-cultures (3), HBMEC + astrocyte co-cultures, and (4) HBMEC + pericyte + astrocyte co-cultures robustly formed perfusable cerebral networks. Co-culture conditions showed stratified organization of pericytes, positioned between endothelial cells and astrocytes, and astrocytes ensheathing vessels, exhibiting direct contact verified through immunocytochemistry characterization. Although the vessels were not continuously covered by astrocytic endfeet, the morphological relationships between endothelial cells, pericytes and astrocytes are comparable to the blood brain barrier structure in vivo [37, 38]. This structural mimicry is essential, as both cell types are known to regulate BBB integrity, basement membrane integrity, and BBB-specific gene expression through paracrine signaling and physical contact [39–41]. HBMEC monocultures and co-cultures of HBMECs and astrocytes presented with higher permeability scores, larger vessel diameters, and fewer branches compared to co-cultures containing pericytes. Addition of pericytes resulted in lower permeability scores, smaller vessel diameters, and more branching. These effects were most pronounced in tricultures containing all three cell types. Together, these data show more intricate microvascular network formation in presence of astrocytes and pericytes.

Furthermore, the inclusion of pericytes – in absence or presence of astrocytes – resulted in vascular structures that were more aligned in the direction of flow. This observation suggests that pericytes may play a critical role in guiding endothelial remodeling and axial alignment in response to mechanical cues, consistent with in vivo studies highlighting pericyte involvement in vascular development, maturation and stabilization [42, 43]. The observed pronounced effects in the tricultures may also suggest a role of astrocytes in vascular organization by ensheathing blood vessels and regulating cerebral flow although their function is context-dependent as it is influenced by developmental-maturational stage, interactions with other cell types and pathological conditions [44–46]. One must note that the cell ratios used in this study were selected based on in vitro optimization, focusing on recapitulating key in vivo BBB characteristics including microvascular network formation, lumen development, barrier function, and expression of relevant markers and was prioritized over retaining in vivo-like cell ratios. Additionally, cell ratios were not equal for astrocytes (500 cells/µL) and pericytes (2,500 cells/µL), complicating direct comparison of HBMEC + astrocyte and HBMEC + pericyte co-cultures.

Several other reports previously described in vitro BBB co-culture models in Transwell or the formation of self-assembling BBB vascular networks in fibrin-based ECM gels [22, 23, 47, 48]. There is great promise in creating complex disease models in Organ-on-a-chip systems, but these are often limited by the use of external pump systems and a lack of throughput [49]. Gravity driven perfusion has been used to address this limitation allowing for a scalable system with an open well architecture which aids the ease of use, but the downside is a non-physiological bidirectional flow. Gravity driven systems providing unidirectional flow have been reported in literature, but are typically limited in throughput [50–54]. Very recently, Rajput et al. described a self-assembling BBB vascular network model in the OrganoPlate^®^ Graft using brain endothelial cells and pericytes to study the effect of Flavivirus on BBB dysfunction [55]. The model described in the current manuscript builds onto this work adding amongst others unidirectional flow instead of bidirectional fluid flow through the microvasculature.

The unidirectional flow may allow for improved assessment of vascular function, as flow disturbances have been shown to induce vascular dysfunction in vivo [56]. Our simple yet physiologically relevant setup supports continuous perfusion through the lumenized vascular network, making it cost-effective, scalable, and well-suited for studying immune cell trafficking and drug permeability under dynamic flow conditions. Furthermore, our work incorporates astrocytes alongside pericytes and endothelial cells, enabling further research into pericytes’ and astrocytes’ distinct and combined contributions to BBB function in health and disease.

Despite its advantages, this BBB model is subject to limitations that should be considered. First, the culture conditions could be further improved by the addition of astrocyte supplements to the media to support the astrocytes health in the HBMEC-astrocytes co-culture. This becomes less crucial upon addition of pericytes, suggested by their ability to secrete transforming growth factor-β (TGF-β) and direct cell-cell interactions [38, 57]. Second, potential concerns with the use of primary cells include a lack of consistency due to donor differences or loss of cells’ characteristic features upon culture in vitro [58]. Third, the models presented here predominantly rely on fibrin as an ECM gel, similar to models reported by others [22, 23]. Given that increased fibrin deposition is a hallmark of stroke and neuroinflammation, its presence may limit the suitability of these models for representing a healthy, homeostatic neurovascular unit [59, 60]. Future work may benefit from evaluation of alternative ECM gels. Fourth, this study assessed the BBB vascular networks’ ability to retain fluorescent dyes as a measure of barrier function. A permeability score that reflects the fluorescent dye signal outside and inside the vascular network showed significant differences between different mono- and co-culture setups. However, due to the single timepoint nature of the assay, no standardized measure of permeability such as P_app_ was provided. Therefore, the barrier function of the models reported here cannot be directly compared to other approaches. In addition, employing dextran molecules with varying molecular weights under prolonged incubation are required for in-depth characterization of the models’ barrier function. Finally, while a proof-of-concept experiment indicates P-gp activity in the present models, comprehensive characterization of transporter expression and functionality is necessary to evaluate their suitability for transport studies.

The model presented here may be applied to study common neurological diseases, such as stroke, using an in vitro approach that mimics reduced perfusion and oxygen deprivation [35]. Additionally, the gravity-based perfusion flow is well suited for assessing neuroinflammatory adhesion and intra- or extravasation of circulating immune cells such as leukocytes into the brain compartment [61, 62]. While the current cerebral vasculature model incorporates several key cellular structures of the human BBB, the inclusion of additional cell types involved in the neurovascular unit (NVU), such as neurons and microglia, would further improve the physiological relevance of the model [63–65].

## Conclusion

We present a 14-day stable, highly robust self-assembling BBB co-culture model containing primary human brain endothelial cells, pericytes, and astrocytes. This model allows for assessment of each cell type’s distinct contribution as well as their combined effects. With 48 chips in one plate and gravity driven unidirectional flow through a cerebral vascular network, this model poses a scalable and applicable tool in the study of BBB dysfunction and restorative therapies.

## Supplementary Information

Below is the link to the electronic supplementary material.

Supplementary Material 1

Supplementary Material 2

Supplementary Material 3

Supplementary Material 4

Supplementary Material 5

Supplementary Material 6

Supplementary Material 7

Supplementary Material 8

Supplementary Material 9

## Data Availability

The datasets used and/or analyzed during the current study are available from the corresponding author on reasonable request.

## Abbreviations

AQ4: Aquaporin 4
BBB: Blood-brain barrier
CNS: Central nervous system
ECM: Extracellular matrix gel
GFAP: Glial fibrillary acidic protein
HBMEC: Human brain microvascular endothelial cell
NVU: Neurovascular unit
PECAM-1: Platelet endothelial cell adhesion molecule
PDGFRβ: Platelet derived growth factor receptor beta
P-gp: P-glycoprotein
TGF-β: Transforming growth factor-β
VE-cadherin: Vascular endothelial cadherin
UF: Uniflow
