# Supplementary material for: Building the blood-brain barrier: a scalable self-assembling 3D model of the brain microvasculature under unidirectional flow
Source: Fluids Barriers CNS. 2026 Jan 23;23:29. doi: 10.1186/s12987-026-00765-x (PMC12910996; doi:10.1186/s12987-026-00765-x)
Supplement: Supplementary file 1 — Supplementary Material 1 [file 12987_2026_765_MOESM1_ESM.docx]

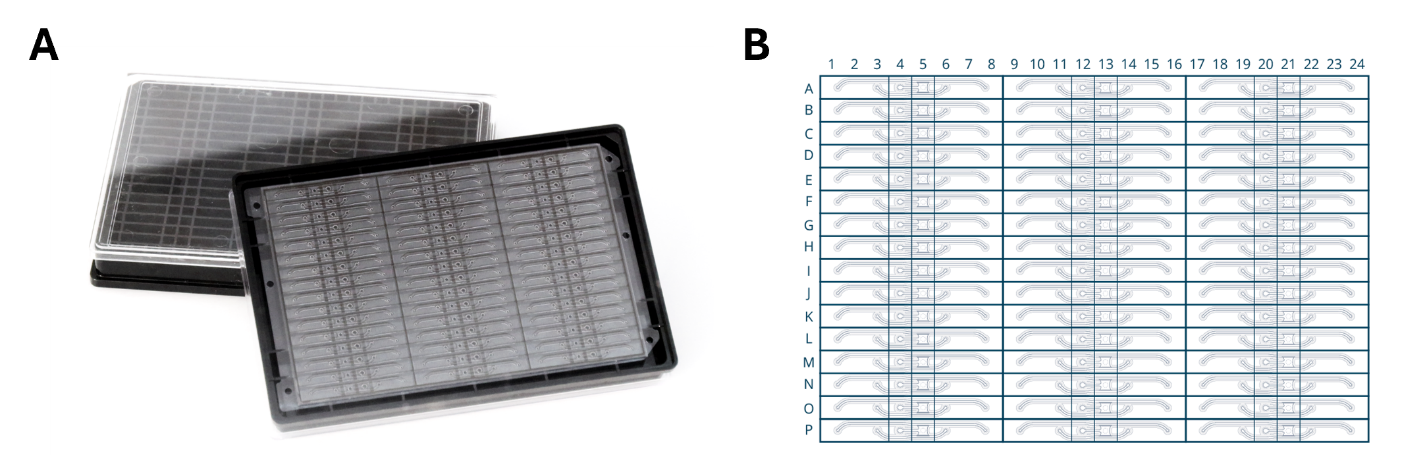


**Supplementary Figure 1 | The OrganoPlate Graft 48 UF.** **(A)** Top and bottom view of the OrganoPlate Graft 48 UF. **(B)** Plate layout of the OrganoPlate Graft 48 UF showing 48 individual tissue culture chips in the format of a modified 384-well culture plate.


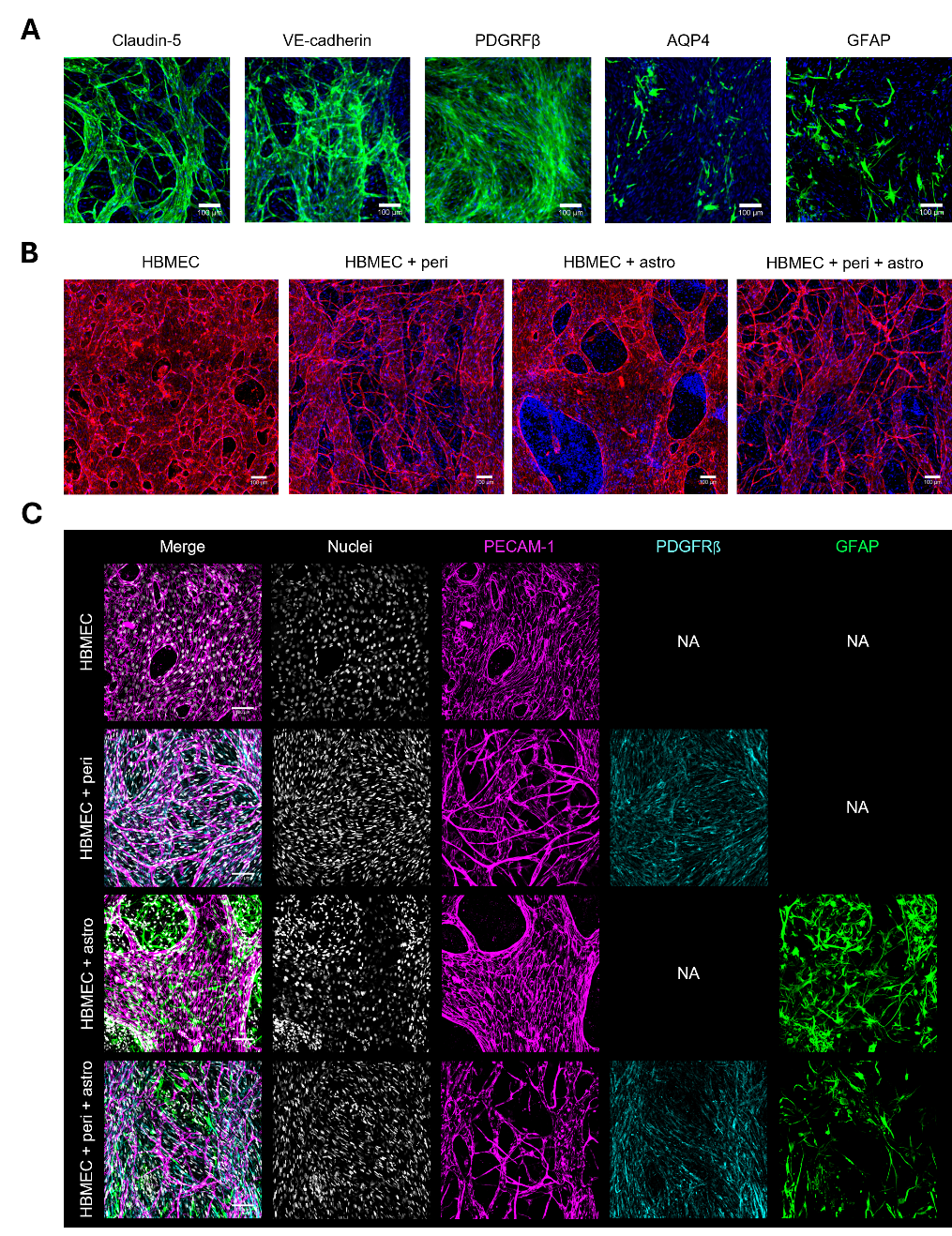


**Supplementary Figure 2 | Expression of endothelial, pericytic, and astrocytic markers. (A)** Co-cultures of HBMEC, astrocytes, and pericytes were fixed at day 14. HBMECs were stained for tight junction protein Claudin-5 and adherens junction protein VE-cadherin. Pericytes were stained for PDGFRβ and astrocytes were stained for AQP4 and GFAP. Scale bar = 100 µm. **(B)** Co-cultures of HBMEC, astrocytes, and pericytes were fixed at day 14 and stained for endothelial marker PECAM-1. Scale bar = 100 µm. PECAM-1 staining images were used for vascular network quantification shown in figure 2C-F. **(C)** High magnification maximum projection images of the nuclear marker (gray), endothelial cell marker PECAM-1 (magenta), pericytic marker PDGFRβ (cyan), and astrocytic marker GFAP (green) for the different BBB culture setups. Not applicable (NA) in absence of cell type. Scale bar = 100µm.


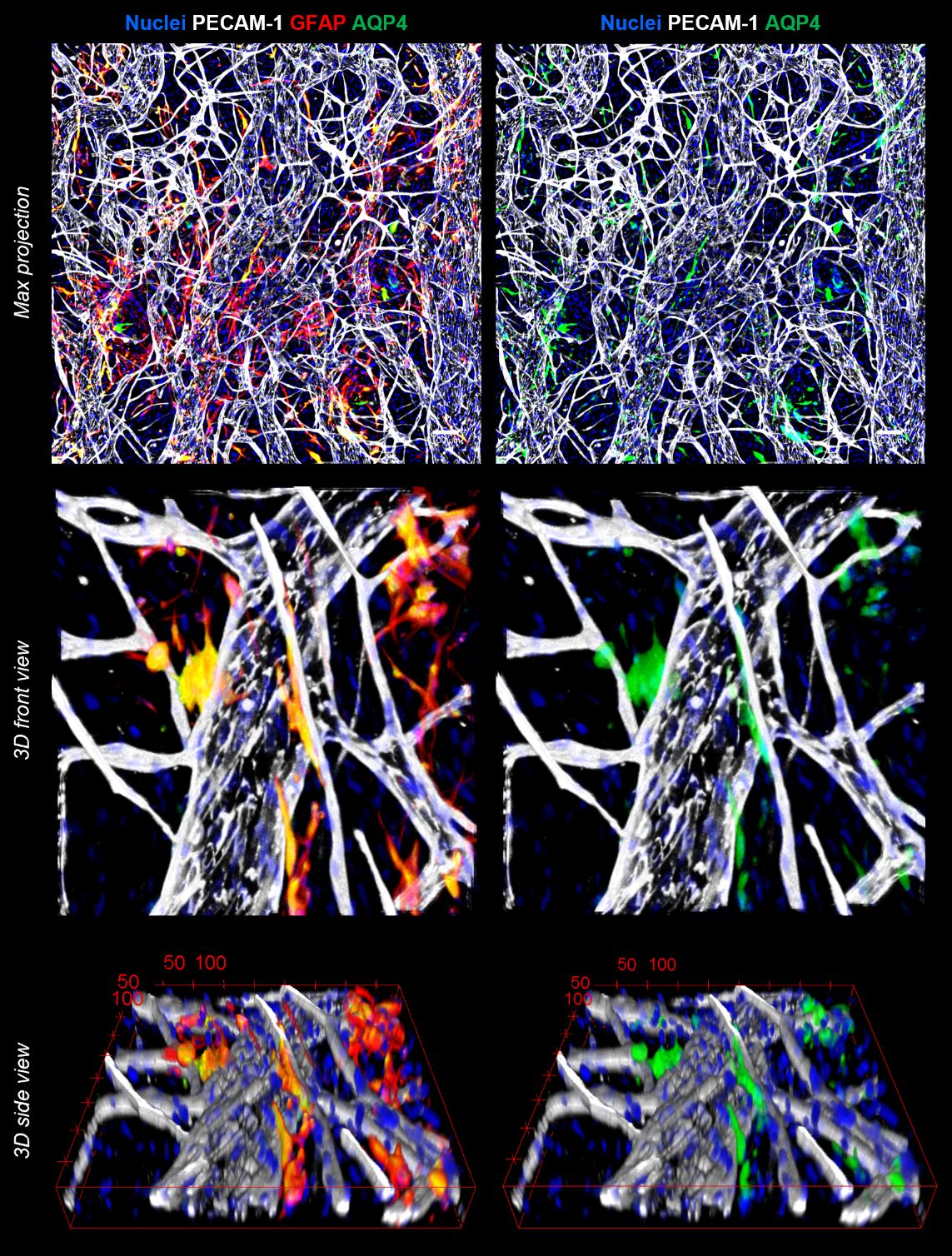


**Supplementary Figure 3 | Astrocytic endfeet and endothelial vessel interaction confirmed by aquaporin-4 expression.** Co-culture of HBMEC, astrocytes, and pericytes were stained for nuclei (blue), PECAM-1 (gray), AQP4 (green) and GFAP (red). Images depicted as maximum projections and 3D reconstructions (front and side views). Scale bar = 100µm


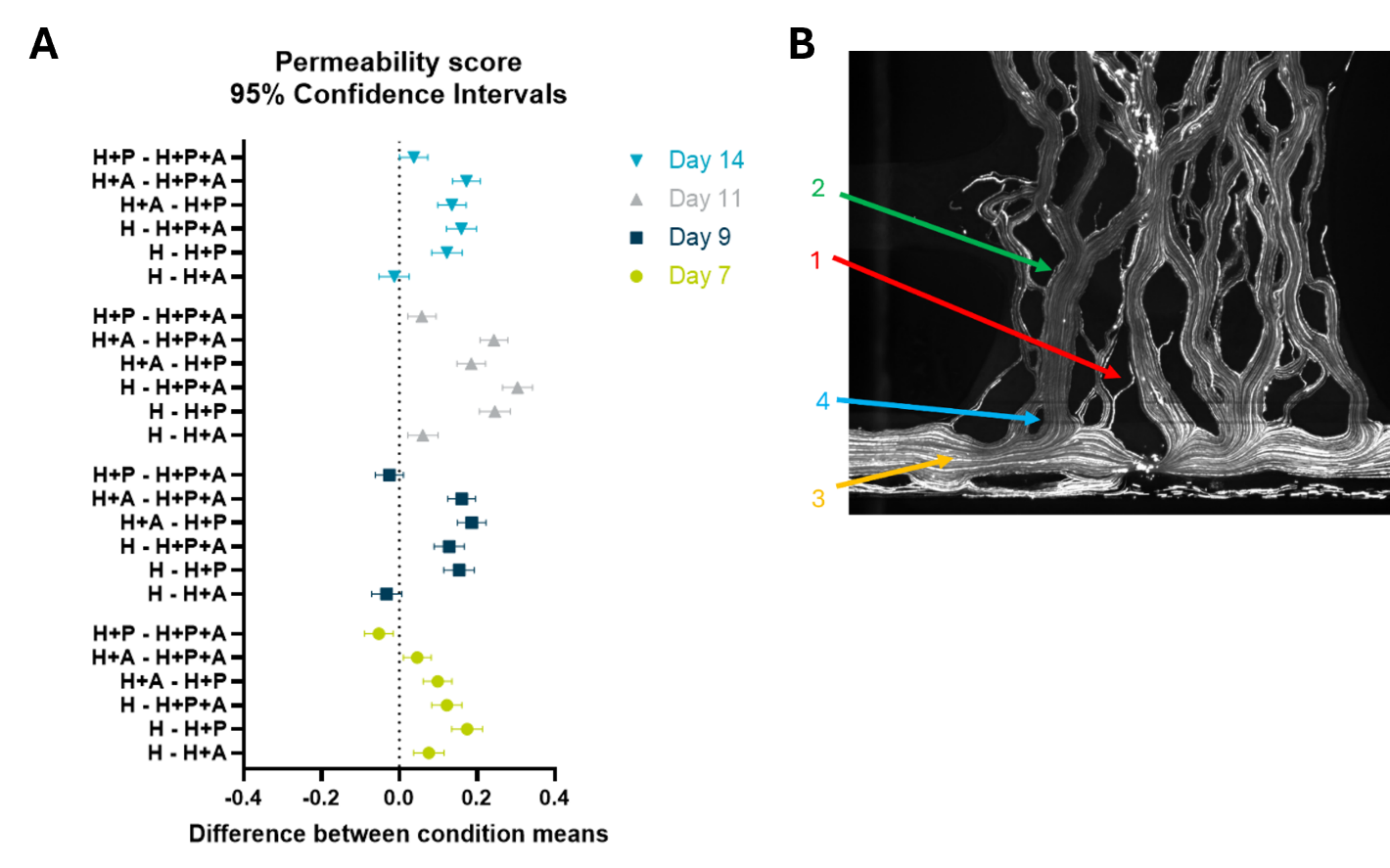


**Supplementary Figure 4 | Statistical analysis of permeability data and flow speed visualization. (A)** A two-way ANOVA was performed on the data presented in main Figure 3C (permeability score for 150 kDa FITC-dextran dye in different culture setups at different days). This graph displays the 95% confidence intervals of pairwise differences between conditions. The dotted line represents zero difference between means. Confidence intervals that do not overlap with this line indicate statistically significant differences, with greater distances from the dotted line reflecting larger differences between conditions. **(B)** Bead path-trace of 2 seconds of the co-culture of HBMECs, pericytes and astrocytes on day 9, visualizing a small vessel (1), a large vessel (2), the main perfusion tubule (3), and its connecting branch (4).

**
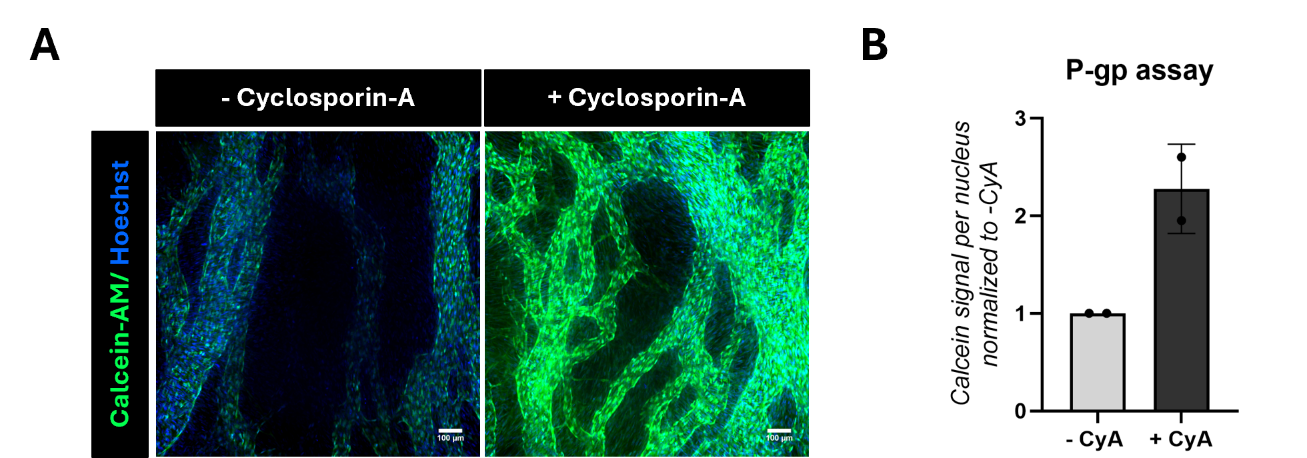
**

**Supplementary Figure 5 | P-gp functionality. (A)** Calcein-AM, a substrate of efflux transporter P-gp, was perfused through co-culture of HBMECs, pericytes, and astrocytes on day X of culture. Calcein-AM was taken up by the cells and converted to green-fluorescent calcein. **(B)** P-gp’s ability to efflux calcein out of the cell was inhibited using cyclosporin-A, resulting in a 2-fold increase in intracellular fluorescence. n = 2 chips. Graphs show mean ± standard deviation.

**Supplementary video 1-4** **| Three dimensional structure of lumenized BBB vascular networks.** Z-slice videos of different BBB vascular bed from bottom to top stained for the nuclear marker (gray), endothelial cell marker PECAM-1 (magenta), pericytic marker PDGFRβ (cyan), and astrocytic marker GFAP (green). Scale bar = 100µm

**Supplementary video 5-8 | Unidirectional bead flow through BBB vascular networks.** Fluorescent beads (1-5 µm size) were perfused through the cerebral vascular networks. Bead flow through vascular networks consisting of (1) HBMEC, (2) HBMEC + pericytes, (3) HBMEC + astrocytes, and (4) HBMEC + astrocytes + pericytes was captured via high-speed fluorescent imaging.
